# Exploring Extracellular Vesicles of Probiotic Yeast as Carriers of Biologically Active Molecules Transferred to Human Intestinal Cells

**DOI:** 10.3390/ijms241411340

**Published:** 2023-07-12

**Authors:** Jolanta Mierzejewska, Patrycja Kowalska, Klaudia Marlicka, Sara Dworakowska, Ewa Sitkiewicz, Maciej Trzaskowski, Agata Głuchowska, Grażyna Mosieniak, Małgorzata Milner-Krawczyk

**Affiliations:** 1 Chair of Drug and Cosmetics Biotechnology, Faculty of Chemistry, Warsaw University of Technology, 00-664 Warsaw, Poland; patrycja.kowalska3.dokt@pw.edu.pl (P.K.); klaudia.marlicka.dokt@pw.edu.pl (K.M.); sara.dworakowska.stud@pw.edu.pl (S.D.); malgorzata.krawczyk@pw.edu.pl (M.M.-K.); 2 Mass Spectrometry Laboratory, Institute of Biophysics and Biochemistry, Polish Academy of Science, 02-106 Warsaw, Poland; ewa@ibb.waw.pl; 3 Center for Advanced Materials and Technology, Warsaw University of Technology, 02-822 Warsaw, Poland; maciej.trzaskowski@pw.edu.pl; 4 Nencki Institute of Experimental Biology, Polish Academy of Science, 02-093 Warsaw, Poland; a.ciolko@nencki.edu.pl (A.G.); g.mosieniak@nencki.edu.pl (G.M.)

**Keywords:** extracellular vesicles, EVs, probiotic yeast, *Saccharomyces boulardii*, carriers, bioactive molecules, drug delivery system

## Abstract

Extracellular vesicles (EVs) are nanoparticles containing various bioactive cargos—e.g., proteins, RNAs, and lipids—that are released into the environment by all cell types. They are involved in, amongst other functions, intercellular communication. This article presents studies on EVs produced by the probiotic yeast *Saccharomyces boulardii* CNCM I-745. The size distribution and concentration of EVs in the liquid culture of yeast were estimated. Moreover, the vesicles of *S. boulardii* were tested for their cytotoxicity against three model human intestinal cell lines. This study did not show any significant negative effect of yeast EVs on these cells under tested conditions. In addition, EVs of *S. boulardii* were verified for their ability to internalize in vitro with human cells and transfer their cargo. The yeast vesicles were loaded with doxorubicin, an anticancer agent, and added to the cellular cultures. Subsequently, microscopic observations revealed that these EVs transferred the compound to human intestinal cell lines. A cytotoxicity test confirmed the activity of the transferred doxorubicin. Detailed information about the proteins present in EVs might be important in terms of exploring yeast EVs as carriers of active molecules. Thus, proteomic analysis of the EV content was also conducted within the present study, and it allowed the identification of 541 proteins after matching them to the Saccharomyces Genome Database (SGD). Altogether, this study provides strong evidence that the EVs of the probiotic CNCM I-745 strain could be considered a drug delivery system.

## 1. Introduction

Cells, whether eukaryotic or prokaryotic, can secrete membrane vesicles filled with various types of molecules, e.g., proteins, DNA, and RNA. They use these phospholipid structures to communicate with other cells, respond to changes in the surrounding environment, or obtain food [1]. From research on pathogenic microscopic fungi—mainly *Candida albicans* and *Cryptococcus neoformans*—it is known that these microorganisms, after infecting the human body, secrete EVs that are necessary for effective host colonization [2,3]. Since 2007, when *C. neoformans* was proven to secrete EVs into the environment, similar studies have been published for 25 other yeast species, mostly pathogenic [4]. Reports show that yeast EVs, similar to the best-known mammalian EVs, are membrane vesicles with sizes mainly in the range of 20–400 nm. In addition to proteins, they contain other molecules, e.g., miRNA or mRNA, and are most likely responsible for communication between different cells in the environment [5]. However, much more research is required to understand both the factors determining the charge composition of EVs and the mechanisms of their secretion by yeast. Furthermore, it is also unknown how yeast EVs interact with other cells, whether human, animal, or plant. This is because extracellular vesicles are not a homogeneous group of molecules and have sizes on the order of nanometers; so, their isolation and study is a challenge [6].

Knowledge about the EVs produced by GRAS (generally recognized as safe) yeast present in fermented food is even more limited than that of pathogenic fungi. To date, there have been publications showing that the classic yeast *Saccharomyces cerevisiae* or so-called unconventional wine-making yeasts such as *Torulaspora delbrueckii* or *Metschnikowia pulcherrima* secrete extracellular membrane vesicles containing a variety of proteins [7]. Therefore, it can be assumed that EV production and secretion among different yeast species are common, although this needs to be verified experimentally. We are convinced that probiotic microorganisms should be included in this kind of research because they are increasingly used as a dietary supplement.

Probiotics have a well-established positive effect on the functioning of our body by stabilizing the autochthonous microbiome, protecting against colonization by pathogens, and secreting enzymes that help digest food as well as substances that stimulate the host’s immune system [8]. Lactic acid bacteria are mainly among them; however, yeasts, e.g., from the genera *Saccharomyces* and *Kluyveromyces*, are also present. To date, the best documented probiotic effectiveness in clinical trials has been shown by the yeast *Saccharomyces boulardii* CNCM I-745 [9,10,11,12,13]. This raises the question of whether the probiotic activity of the microorganisms we consume with food or dietary supplements is related to the EVs they secrete. In the case of lactic acid bacteria, recent reports indicate that such a correlation is possible [14]. Notably, in 2022, one publication showed that EVs released by *S. cerevisiae* activated the immune response of mouse macrophage-like and dendritic cells [15]. Since *S. boulardii* is morphologically and physiologically remarkably similar to *S. cerevisiae* and has recently been recognized as its subspecies by some microbiologists, its EVs may likely act similarly [16]. However, this needs to be confirmed by research. Moreover, it would be interesting to study whether probiotic yeast can transfer proteins, nucleic acids, and other biomolecules via EVs into human gastrointestinal cells and exert any influence on their metabolic activity. Assuming that such a hypothesis is true, in the future, it would be possible to create genetically modified probiotic yeast strains that would secrete in the intestinal tracts, for example, recombinant proteins with therapeutic or immunizing effects in the vesicles. This study aimed to verify whether EVs of *S. boulardii* CNCM I-745 can be considered drug carriers. The research was divided into three main tasks: (1) isolation and morphological and proteomic characterization of EVs from yeast cultures; (2) study of changes in the metabolic activity of model human intestinal cell lines in the presence of yeast EVs; and (3) investigation of whether EVs are able to effectively transfer their cargo to human cells.

## 2. Results and Discussion

### 2.1. Characterization of EVs Produced by S. boulardii CNCM I-745

**Figure 1:**
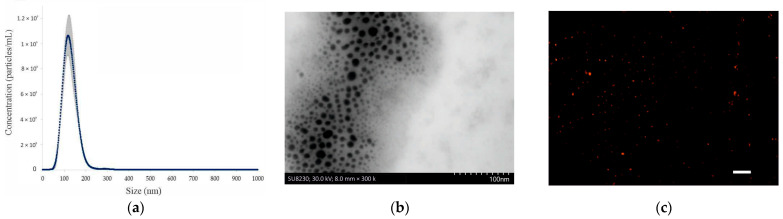
Characteristics of EVs produced by *S. boulardii* CNCM I-745. (**a**) Concentration and size distribution of EVs in the samples determined by NTA; mean values (blue line) ± SD (gray lines); *n* = 3. (**b**) STEM micrograph of isolated EVs; lipid structures are in black because of the use of the reverse staining technique. (**c**) Nile Red stained EVs visualized (red dots) by fluorescence microscopy (scale bar 10 μm).

The first step of the study was to obtain extracellular vesicles produced by *S. boulardii* CNCM I-745. Therefore, yeast cultures were grown in standard YPD liquid medium; then, suspensions of EVs in PBS were obtained using sequential centrifugation, filtration, and ultrafiltration. The size and concentration of EVs in the samples were determined using nanoparticle tracking analysis (NTA). NTA revealed that most of the nanoparticles were in the size range between 30 and 230 nm with a mean value of 127.39 ± 1.34 nm (Figure 1a). Similarly, it has been recently reported for *S. cerevisiae* that it produced EVs with sizes between 25 and 250 nm [15]. Nevertheless, it should be noted that one of the first steps in the purification of EVs was the removal of yeast cells by routine filtration on a membrane with 0.2 µm pores, which significantly eliminated particles larger than 200 nm from the purified mixture. Therefore, it cannot be completely ruled out that the CNCM I-745 strain also secretes bigger EVs. Based on the number of EVs in the samples assessed via NTA (8.20 ± 1.12 × 10^10^ EVs/mL), an EV concentration of 5.49 × 10^9^ EVs/mL in the 150 mL cultures of *S. boulardii* in YPD medium was calculated. For comparison, the fungus *Aureobasidium pullulans* produced 6.10 × 10^8^ EVs/mL of minimal YNB (yeast nitrogen based with 2% glucose) culture medium [17]. Furthermore, by knowing the number of viable cells in the cultures of *S. boulardii* (1 × 10^8^ cfu/mL), it was also possible to determine that a single yeast cell released approximately 55 EVs. A similar number of 44 EVs per cell was recently reported for the pathogenic yeast *Candida tropicalis* [18]. No such data were found for *S. cerevisiae*. Although these are only estimates, as it is not known how many EVs are lost during isolation from the culture, they provide some insight into the efficiency of vesicle production by *S. boulardii*, which is important when considering these nanoparticles as carriers of biologically active substances.

Next, the yeast vesicles were visualized both by scanning transmission electron microscopy (STEM) (Figure 1b) with the reverse staining method and by fluorescence microscopy after staining EVs with Nile Red, which binds to lipids in membranes (Figure 1c).

These two applied techniques allowed us to confirm that lipid nanostructures from probiotic yeast culture were successfully isolated.

### 2.2. Metabolic Activity Tests on Human Intestinal Cell Lines Treated with the EVs of Probiotic Yeast

Considering that yeast strain CNCM I-745 is a probiotic, it can be assumed that its EVs should not also negatively affect the metabolism of mammalian cells, although no one has yet investigated this. Therefore, after isolation and characterization, *S. boulardii* extracellular vesicles were tested in vitro for cytotoxicity against human cells. For this purpose, model cell lines derived from colorectal tissues, two cancerous cell lines (HT-29 and HCT116), and one normal cell line (CCD841 CoN), were exposed to various concentrations of EVs for 24 h. It should be emphasized that the EV suspensions were prepared in such a way so as to maintain the appropriate proportion of the number of EVs in relation to mammalian cells, i.e., 1.8 × 10^3^, 1.8 × 10^4^, 9.2 × 10^4^, and 1.8 × 10^5^ EVs per single human cell.

**Figure 2:**
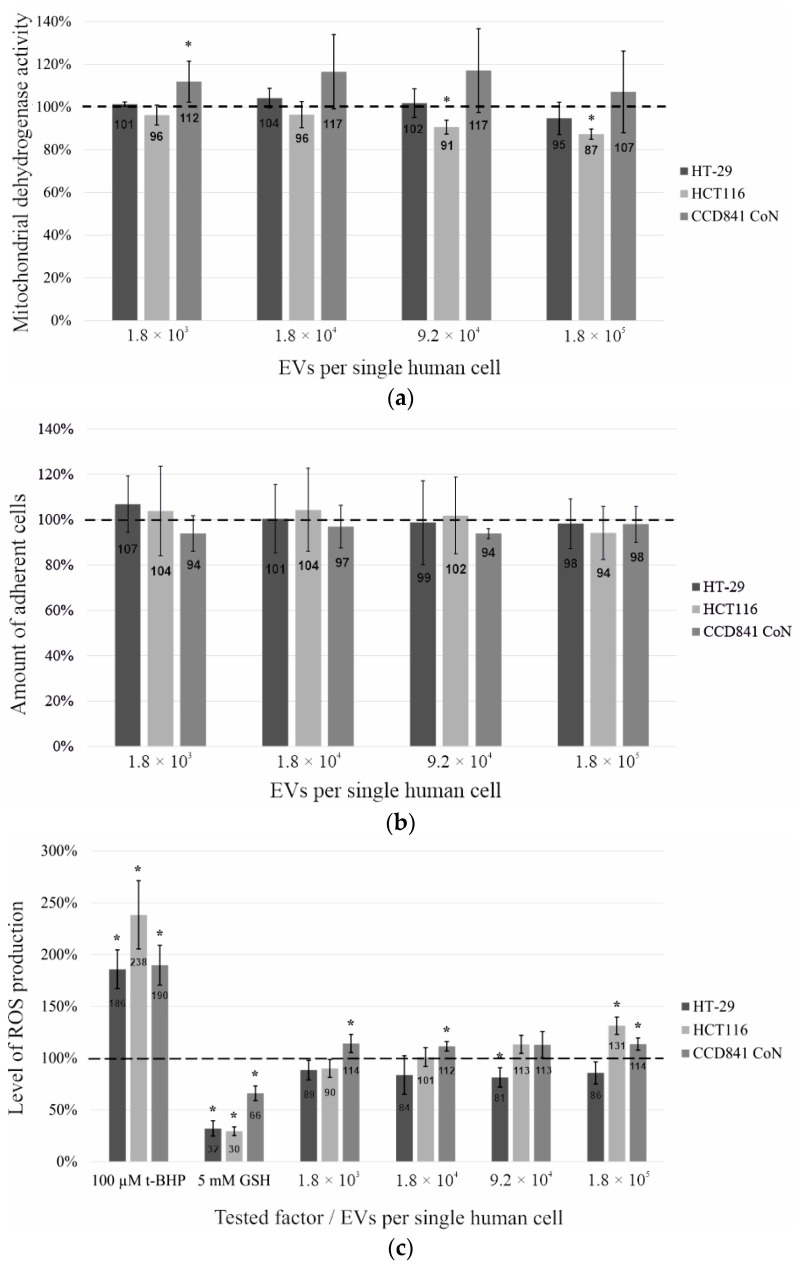
The effect of EVs of probiotic yeast on the metabolic activity of two model human intestinal cancer cell lines (HT-29 and HCT116) and one normal cell line (CCD841 CoN). (**a**) MTT assay—measurement of mitochondrial dehydrogenase activity, a basic cytotoxicity test. (**b**) Crystal violet assay—determination of the number of adherent cells, which can be correlated with the number of viable cells. (**c**) ROS assay—estimation of the level of reactive oxygen species production; two additional controls were applied, oxidant (t-BHP, tert-butyl hydroperoxide) and antioxidant (GSH, glutathione), to confirm the proper functionality of the assay. Each tested variant was compared with the nontreated control cells (100%, marked by a dashed line) and presented as a percent of the control activity level; mean values ± SD from three independent biological experiments performed in triplicate; * statistical significance in comparison with the nontreated controls, *p*-value < 0.05.

After 24 h of exposure to the yeast vesicles, the cell lines were subjected to two routine metabolic assays to check the viability of the cells. First, an MTT assay, based on the ability of mitochondrial dehydrogenase to convert yellow tetrazolium salt into purple formazan, was applied (Figure 2a). Here, only one cell line, HCT116, showed a slight (9–13%) decrease in the activity of this enzyme at the two highest concentrations of EVs. The other two cell lines did not show reduced viability, and a slight increase (7–17%) was even observed for the CCD841 CoN line. In the next test, the effect of EV presence on the adherence of intestinal cells was checked via crystal violet assay (Figure 2b). No significant changes in the number of adherent cells were observed, which also confirms that the 24 h incubation with yeast EVs did not adversely affect the viability of the tested cells.

The third approach was used to test whether the presence of yeast EVs enhances the increased production of reactive oxygen species (ROS) in intestinal cells. This assumption needs to be verified, as it is known that the long-term increased level of ROS can lead to oxidative stress and, consequently, pathological changes [19,20].

The present study showed that the HT-29 and CCD841 CoN cell lines produced ROS at the same level regardless of the EV concentration (Figure 2c). However, a slight (12–14%) increase in ROS was noticed for the CCD841 CoN line in comparison with the control untreated cells. However, the second tumor line HCT116 significantly increased (31%) ROS secretion only in the presence of the highest EV concentration. It has frequently been reported that, although isolated from colorectal cancer, the two cell lines HT-29 and HCT116 show some phenotypic differences. For instance, HCT116 is a highly aggressive cell line that shows no ability to differentiate, while HT-29 has an intermediate capacity to differentiate [21]. Thus, it is not surprising that the two tumor lines may react slightly differently to the presence of yeast EVs. The higher level of ROS in the case of HCT116 cells can also be correlated with its greater sensitivity in the MTT assay, as ROS are one of the basic signaling molecules during the induction of apoptosis in the mitochondrial pathway [22].

Based on EN ISO 10993-5, a cytotoxic effect is considered only when the cell viability is reduced by more than 30%. Considering this, it can be concluded that 24 h exposure of the tested model intestinal lines to the EVs of probiotic yeast is not cytotoxic and thus might be considered as drug carriers. However, the slightly reduced viability and the increase in ROS production in the highly aggressive tumor cell line HCT116 is also intriguing, which may indicate the potential anticancer effect of EVs from probiotic yeast.

### 2.3. Determination of Whether Extracellular Vesicles of Probiotic Yeast Can Transfer Biologically Active Substances into Human Cells

**Figure 3:**
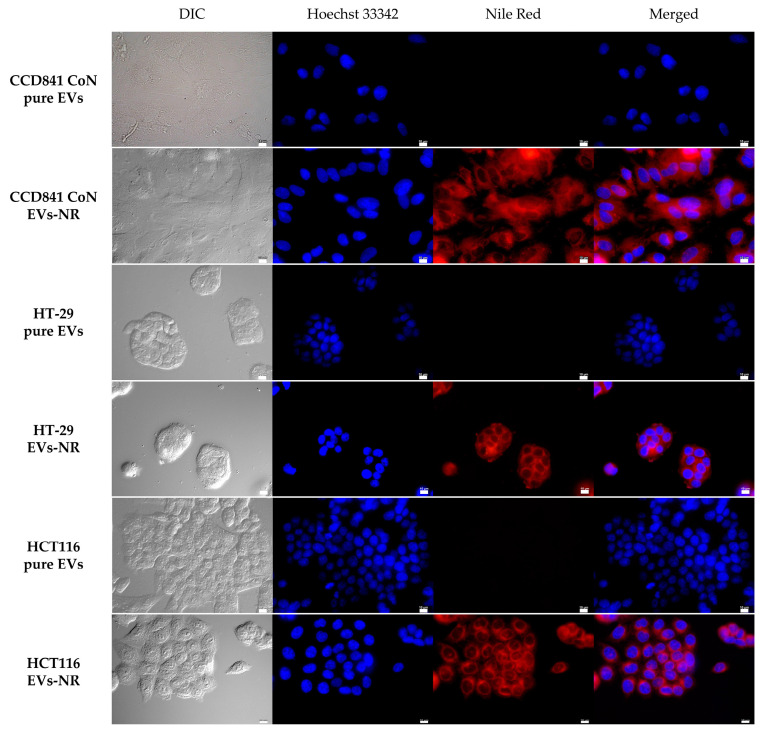
Internalization of *S. boulardii* EVs with human intestinal cell lines CCD841 CoN, HT-29, and HCT116. EVs-NR—vesicles stained with red-fluorescence Nile Red dye; pure EVs as a control; EVs were added to the cell cultures at a concentration of 4.5 × 10^4^ EVs/cell; cellular nuclei stained with Hoechst 33342 as a reference point; scale bar, 10 µm.

Having obtained evidence that the extracellular vesicles of probiotic yeasts were not cytotoxic, the next task was to test whether *S. boulardii* vesicles were capable of transferring their cargo to human cells. First, however, it was necessary to verify whether the yeast EVs internalize with cells. For this purpose, lipid bilayers of EV membranes were stained with a lipophilic dye, red-fluorescence Nile Red, and subsequently incubated for 24 h with HT-29, HCT116, and CCD841 CoN cells. Afterwards, the cells were fixed and stained with Hoechst 33342, and microscopic observations were conducted. The cells incubated with the EVs stained with Nile Red showed red fluorescence originating from the lipophilic dye. The signal was mainly localized in the cell membrane and cytoplasm, indicating that the EVs of *S. boulardii* had entered the human cells (Figure 3). Although extracellular vesicles from probiotic bacteria were proven to be internalized by intestinal epithelial cells in several studies [14], to date, no one has published such an observation for a probiotic yeast.

**Figure 4:**
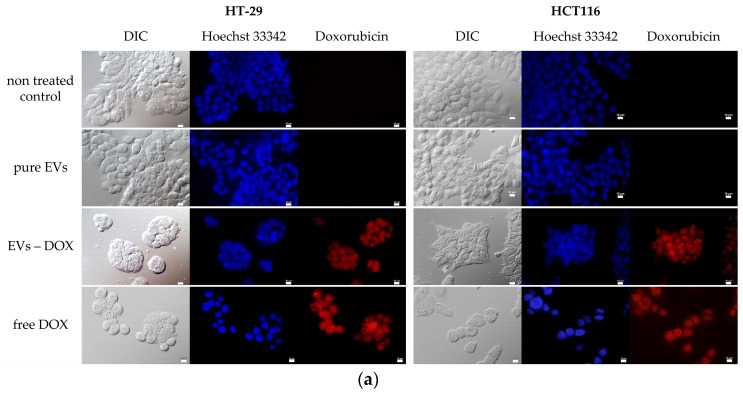

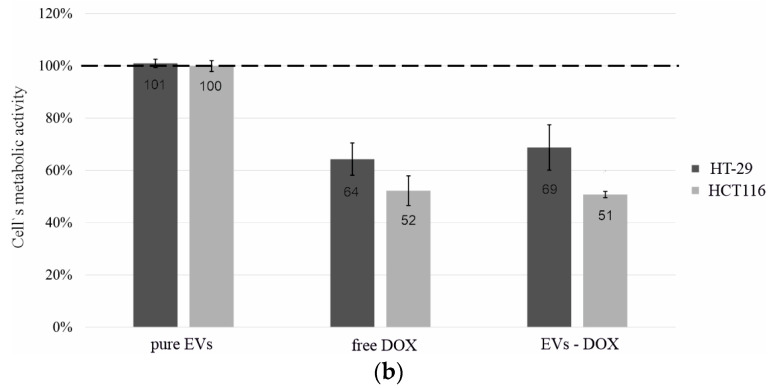
The transfer of doxorubicin (DOX) to the human cell lines HT-29 and HCT116 by EVs of *S. boulardii*. (**a**) Microscopic observations were performed to monitor DOX localization in cells treated with pure EVs, and DOX in PBS solution (free DOX) was applied as a control; DIC (bright field); cellular nuclei stained with Hoechst 33342 dye; scale bar, 10 µm. (**b**) The influence of free DOX and DOX enclosed in EVs (EVs-DOX) on the metabolic activity of the cells (MTT assay); EVs were added to the cell cultures at a concentration of 4.5 × 10^4^ EVs/cell; each tested variant was compared with the nontreated control cells (100%, marked by a dashed line) and presented as a percent of the control activity level; mean values ± SD from three independent biological tests performed in triplicate.

In the subsequent step, doxorubicin (DOX), a hydrophilic antibiotic with antitumor activity that shows red fluorescence and binds DNA, was introduced into the yeast vesicles. It was aimed at examining the penetration of EVs loaded with doxorubicin into cells and the effectiveness of the substance transported in this way. For this purpose, the cancer cell lines HT-29 and HTC116 were incubated with the loaded EVs for 24 h. Subsequently, microscopic observations were performed and showed that doxorubicin, both free and encapsulated in EVs, entered the cell nuclei (Figure 4a). Moreover, the apparent decrease in cell density indicated a reduction in cell proliferation.

In addition, an MTT assay was performed to confirm the activity of doxorubicin successfully introduced into the cells via the EVs. The assay revealed that both cell types treated with DOX contained in EVs and in unbound form showed reduced viability (Figure 4b). Similar to the previous experiments, in this case, the HCT116 line showed a stronger response to the administered active agent.

These results confirm the hypothesis that EVs of the probiotic *S. boulardii* can transfer a biologically active substance to human cells in vitro.

### 2.4. Proteins Identified in the EVs of S. boulardii

EVs of pathogenic yeast play important functions as bioactive community regulators as well as mediators in the immune response of the host organisms. This is correlated with the content of these vesicles [1,2,3]. Thus, without knowledge about the molecular composition of *S. boulardii* EVs, the possibility of using them as drug carriers, recombinant proteins, or antigens cannot be considered. Therefore, the final step of the present research was to isolate the proteins from *S. boulardii*’s vesicles and to perform MS analysis combined with the search of peptide databases to identify proteins. As there is no database available for *S. boulardii* proteins, the SGD for *S. cerevisiae* was used. In this way, 746 proteins were identified, 541 of which were represented by at least two peptides in all three biological replicates of EV samples. The list of the proteins is available in Supplementary File: Tables S1 and S2.

**Figure 5:**
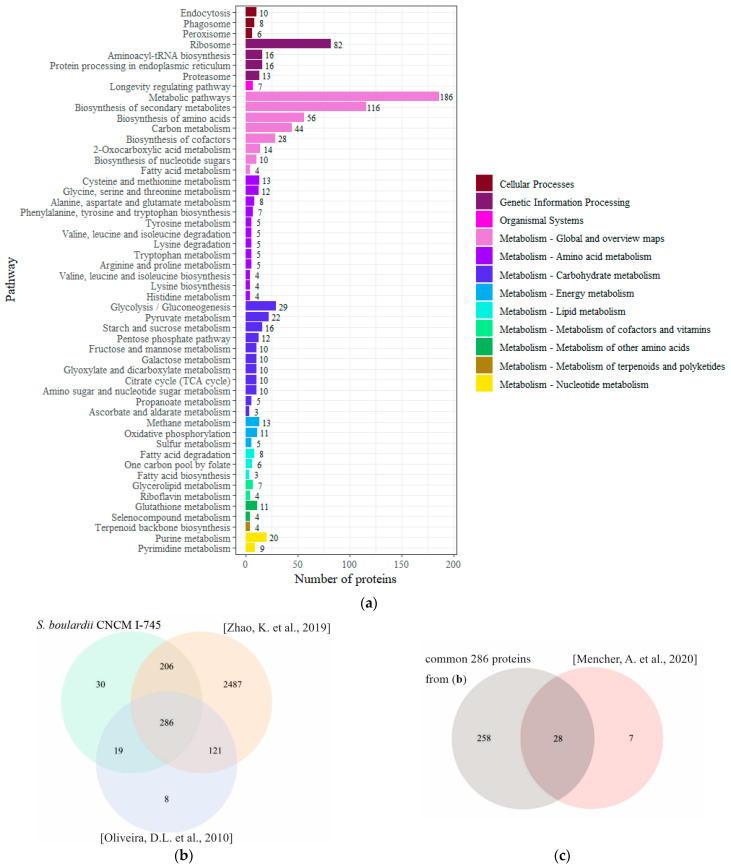
Proteomic analysis of EVs isolated from *S. boulardii* CNCM I-745. (**a**) Functional distribution of proteins identified in the EVs of the CNCM I-745 strain. (**b**) Venn diagram showing similarities of protein composition in the vesicles of *S. boulardii* to two previously reported data for *S. cerevisiae* in [23,24]. (**c**) Venn diagram comparing the 286 common proteins from (**b**) with the proteins reported for *S. cerevisiae* EVs in [7]. The presented results are based on three independent biological experiments.

Some studies on EVs secreted by the yeast *S. cerevisiae* show that these vesicles are rich in proteins that have many functions in cells, mainly in carbon metabolism, cell organization, transport stress response, degradation, and biogenesis of proteins [7,23,24]. The analysis of the identified proteins in *S. boulardii* EVs in terms of their function shows a similar diversity, and as in the *S. cerevisiae* EVs, numerous proteins are involved in basic metabolism (Figure 5a). Moreover, the presence of cytoplasmic metabolic enzymes such as enolase and glyceraldehyde-3-phosphate dehydrogenase or the translation elongation factor has also been reported to be abundant in the vesicles of pathogenic yeast, e.g., *C. albicans* [25]. Thus, it seems to be a tendency; however, it is unclear why yeast EVs are loaded with those proteins.

To gain more information about the proteins frequently present in the EVs of *Saccharomyces* sp., a two-step comparative analysis with three previously published data items from MS analysis of EVs isolated from *S. cerevisiae* was conducted. First, the proteins of *S. boulardii* EVs were compared with the set of 424 proteins identified in [24] and 3100 proteins of [23]. After this analysis, a list of 286 common proteins was obtained (Supplementary File: Table S3, Figure 5b) and subsequently compared with the third set of 35 proteins reported in [7]. Finally, 28 common proteins were selected (Supplementary File: Table S4, Figure 5c). Among these, there are eleven proteins with a function in cell wall organization (e.g., exo-1,3-beta-glucanase, cell-wall-related secretory glycoprotein), eight proteins involved in glycolysis or gluconeogenesis (e.g., pyruvate decarboxylase, enolase), two proteins associated with endoplasmic reticulum (ER) transport/import (protein disulfide isomerase, ATPase), and seven proteins performing other functions (e.g., a mucin family member involved in various signaling pathways, phospholipase B).

Studies on the EVs of pathogenic microscopic fungi show that the composition of the vesicles is dependent on growth conditions, i.e., temperature, culture time, and nutrient availability [5]. Thus, it would be worthwhile in the future to further investigate the protein composition of EVs secreted by probiotic yeasts cultured under yet different conditions. Extensive research in this area will help to determine, among others, the marker proteins for EVs of *S. boulardii*; this, in turn, will enable the development of more precise procedures, based on marker affinity, for the isolation of vesicles from yeast cultures. Besides, knowledge of the molecules often present in EVs could facilitate the tracking of their internalization with human cells and the transfer of these molecules, e.g., by applying immunofluorescence methods.

## 3. Materials and Methods

### 3.1. Yeast Culture for EVs Production

*Saccharomyces boulardii* CNCM I-745 was taken from the sample of ENTEROL (Biocodex, Gentilly, France) and seeded on a standard medium YPD (1% *w*/*v* yeast extract, 2% *w*/*v* bacteriological peptone, and 2% *w*/*v* glucose) solidified with 2% *w*/*v* agar. Then, the single colonies were used to inoculate 10 mL portions of liquid YPD and incubated overnight (approximately 20 h) with a shaking speed of 240 rpm (SI-600R, Lab Companion, Billerica, MA, USA) at 37 °C. The next day, 3 mL of culture was diluted in 150 mL of fresh YPD in a 300 mL flask and incubated for 22 h (220 rpm, 37 °C). Three independent cultures were prepared. After incubation, serial 10-fold dilutions of the cultures were prepared in sterile phosphate-buffered saline (PBS), pH 7.4 (137 mM sodium chloride, 2.7 mM potassium chloride, and 10 mM phosphate buffer, VWR Chemicals, Solon, OH, USA). Then, 100 μL portions of dilutions were spread on agar YPD plates and incubated for 2 days (37 °C), after which the colonies were counted, and the number of colony-forming units per mL of the culture (cfu/mL) was determined. In this way, the concentration of viable yeast cells in liquid cultures was estimated.

### 3.2. Isolation of EVs from Liquid Yeast Cultures

Just after 22 h of incubation, the yeast cultures grown in YPD medium were centrifuged (6000× *g*, 10 min, 4 °C), and the yeast biomass was discarded. The obtained supernatants were filtered through 0.2 μm vacuum filters (PES Filter Unit, VWR International, LLC, Aurora, OH, USA) to remove the residual cell debris. Subsequently, the EVs present in permeates were concentrated on the membrane with a 100 kDa cut-off (Amicon Ultra—15, Ultracel-100, Centrifugal Filters, Merck Millipore Ltd., Burlington, MA, USA) by centrifugation (3200× *g*, 8 min, 4 °C). Then, the EV samples were collected and transferred again into centrifugal filters with a 100 kDa cut-off and washed three times with sterile PBS. Finally, the obtained EV samples were aliquoted and frozen at −80 °C.

### 3.3. Nanoparticle Tracking Analysis (NTA)

The mean size and concentration of EVs were analyzed using a NanoSight NS300 (Malvern Panalytical Ltd., Malvern, UK) equipped with a 488 nm blue laser, as described in [26]. For each measurement, five independent 30 s videos were captured and analyzed using the built-in NanoSight Software NTA 3.2 under the following conditions: temperature, 22 °C; viscosity, 0.947 cP; syringe pump speed, 30 AU; camera type, sCMOS; camera level, 13; frame rate, 24.983 fps, and settings: detection threshold, 5; total frames analyzed, 749. Before measurement, each EV sample was diluted 1:200 (*v*:*v*) in PBS.

### 3.4. Scanning Transmission Electron Microscopy (STEM)

To obtain an overview of the morphology of EV samples, isolated EVs were visualized with a Hitachi SU8230 microscope equipped with a scanning transmission electron microscopy (STEM) detector. The prepared samples were kept at 4 °C for a maximum of 24 h or frozen at −20 °C. Before measurement, the samples were diluted in ddH_2_O 100×, and double staining with UranyLess/lead citrate (DELTA Microscopies, Mauressac, France) was carried out. The 20 µL droplets of the sample, ddH_2_O, UranyLess, and lead citrate were placed on parafilm. The lacey carbon-supported copper grid (Agar Scientific, London, Essex, UK) was placed on each droplet in the following order: 1.5 min incubation on the sample droplet, 1–1.5 min incubation on the UranyLess droplet, 20 s wash on a water droplet, 1 min incubation on Lead citrate droplet, and the final 20 s wash on a water droplet. The grid prepared in this way was air-dried for 1 min and placed in the microscope column. The samples were observed with a SU8230 electron microscope at a working voltage of 30 kV.

### 3.5. Nile Red Staining of EVs

One hundred microliters of EVs was mixed with 4 μL of Nile Red (2.5 mg/mL in acetone, Carl Roth GmbH + Co. KG, Karlsruhe, Germany). Simultaneously, a control probe was prepared by mixing 100 μL of PBS with 4 μL of Nile Red. After 15 min of incubation in the dark and at RT, the samples were transferred into centrifugal filters with a 10 kDa cut-off. Next, the samples were washed 6 times with 400 μL of PBS to rinse off the redundant dye. The control probe was needed to monitor whether the entire free Nile Red was washed out from the samples. A small droplet (10 μL) of the finally obtained stained sample was placed on the microscope slide glass and covered with a coverslip. Imaging was taken under a fluorescence microscope (Nikon Eclipse Ni with color camera combined with the software NIS Elements Basic Research version 5.30, Precoptic, Warsaw, Poland) with a red filter block (FF01-474/27 nm Excitation, FF02-525/45 nm Emission), as Nile Red is excited by green light and emits red light, under the magnification of 1000× with oil immersion (Nikon, Plan Apo VC objective lens 100×/1.40 Oil OFN25 DIC N2).

### 3.6. Human Intestinal Cell Lines

Three human cell lines from the American Type Culture Collection (ATCC, delivered by LGC Standards Sp. z.o.o) were used in the present study, two isolated from colorectal cancers—HT-29 (ATCC-HTB-38) and HCT116 (ATCC-CCL-247)—and one derived from normal colon tissue—CCD841 CoN (ATCC^®^ CRL-1790). Cancers were cultured in McCoy’s 5A medium (VWR) with 10% fetal bovine serum (FBS, Gibco, Billings, MT, USA) and 1% penicillin–streptomycin solution (Sigma-Aldrich, Burlington, MA, USA) additives. Normal CCD841 CoN cells were cultured in MEM (VWR) with 10% FBS, 1% penicillin–streptomycin solution, and 1% L-glutamine solution (Gibco) additives. Passaging via trypsinization was performed by washing out cells with PBS (w/o magnesium and calcium, VWR) and incubation with 0.25% trypsin-EDTA (Gibco) for 5–7 min at 37 °C. To perform tests for this research, all 3 cell lines were used between the 10th and 20th passages.

### 3.7. Metabolic Activity Tests—MTT, Crystal Violet, and ROS Assays

To perform the metabolic activity tests (MTT, crystal violet, and ROS assays), HT-29 and HCT116 cells were seeded on a 96-well plate at 5 × 10^3^ cells per well in full McCoy’s 5A medium. Because CCD841 CoN cells are much larger than HT-29 and HCT116 cells, it was decided to decrease the number of cells per well to 2.5 × 10^3^ in full MEM. The cells were incubated for 24 h at 37 °C and 5% CO_2_ (HF90 Heal Force incubator). Then, EV samples were mixed with a fresh culture medium. In order to maintain the appropriate proportion of the number of EVs in relation to mammalian cells, 2 sets of various EV concentrations were prepared: 9.0 × 10^7^, 9.0 × 10^8^, 4.5 × 10^9^, and 9.0 × 10^9^ EVs/mL in McCoy’s 5A medium (for HT-29 and HCT116 cells); 4.5 × 10^7^, 4.5 × 10^8^, 2.3 × 10^9^, and 4.5 × 10^9^ EVs/mL in MEM medium (for CCD841 CoN cells). For MTT and CV assays, the culture medium was changed on prepared EV suspensions (100 µL/well), and cells were cultured for an additional 24 h under the same conditions. For the ROS assay, there was an extra step described in the below section for this test.

*MTT assay*. After the 24 h incubation of the cells with EV solutions, the medium was discarded, the cells were gently washed with PBS, and 100 µL of McCoy’s 5A with 0.5 mg/mL MTT (3-(4,5-dimethyl-2-thiazolyl)-2,5-diphenyl-2H-tetrazolium, Sigma-Aldrich) or MEM with 1.0 mg/mL MTT was added into each well. The cells were incubated with MTT salt under standard conditions for 2 h. Next, the medium was discarded, and each well was gently washed with PBS. On each well, 50 μL of pure DMSO (dimethyl sulfoxide, POCH) was added and incubated for 5 min at RT on a laboratory cradle. The absorbance was read at a wavelength of 570 nm with the use of a Synergy H4 Hybrid plate reader (BioTek Instruments, Winooski, VT, USA).

*Crystal Violet assay*. After the 24 h incubation of the cells with EV solutions, the medium was discarded. The cells were fixed with 3.7% paraformaldehyde (Sigma-Aldrich) for 15 min at RT in the dark and washed with PBS. Next, 50 μL of 0.5% crystal violet stock (Sigma-Aldrich) was added to each well. The cells were incubated with CV for 45–60 min at RT on a laboratory cradle. Next, the dye was discarded, and each well was gently washed three times for 5 min on a laboratory cradle, first with 400 μL of PBS and then twice with 400 μL of dH_2_O. Then, dH_2_O was discarded, and the plate was left to dry at RT. In the last step, 50 μL of methanol was added to each well and incubated for 5 min on a laboratory cradle. The absorbance was read at a wavelength of 570 nm with the use of a Synergy H4 Hybrid plate reader.

*ROS (Reactive Oxygen Species) assay*. The methodology was adapted from previous work [27]. Media from 24 h cultures grown in 96-well plates were discarded, and the cells were gently washed with PBS. One hundred microliters of freshly prepared 20 µM dcFDA (2′,7′-dichlorodihydrofluorescein diacetate; Sigma-Aldrich) solution in McCoy’s 5A or MEM medium was added to each well. The plate was incubated for 1 h in the dark at 37 °C. After that time, the medium with dcFDA was discarded, and the cells were gently washed with PBS. The freshly prepared testing variants, 100 µM t-BHP (tert-butyl hydroperoxide; Sigma-Aldrich), 5 mM GSH (glutathione; Sigma-Aldrich), and EV solution variants (in McCoy’s 5A or MEM medium), were added to a plate at a volume of 100 µL per well. The cells were incubated for an additional 24 h under standard conditions. The next day, the fluorescence intensity was read at 485/535 nm (excitation/emission) with the use of a Synergy H4 Hybrid plate reader.

### 3.8. Loading of EVs with Doxorubicin

Fifty microliters of 4.5 mM doxorubicin (doxorubicin hydrochloride, Pol-Aura, Zabrze, Poland) solution in PBS was added to two probes: 450 μL of EVs and 450 μL of sterile PBS as a control. Then, the samples were kept on ice for 30 min. Next, 500 μL of a 400 mM sucrose solution [28] was added, and the samples were incubated at 4 °C for 20 h. After that, to separate unbound doxorubicin, samples were transferred to filter columns with a cut-off of 10 kDa and centrifuged (15 min, 14,000× *g*). The filtrates were transferred to new tubes, and 400 μL of sterile PBS was applied to the columns to wash away doxorubicin. Then, the samples were centrifuged again, the filtrates were removed, and the washing step was repeated four times until the pink color of doxorubicin was no longer visible in the filtrates, and the fluorescence of doxorubicin was not detected at excitation wavelengths of 480 nm/emission wavelengths of 530 nm, according to [28]. The filter columns were then inverted and placed in new tubes, which were centrifuged (2 min, 1000× *g*) to recover the samples of stained EVs. The final volume of the EV sample and control sample was adjusted to an initial volume of ~400 μL with PBS. Doxorubicin concentration in EVs samples was estimated at 7.5 μM.

### 3.9. Incubation of Human Cells with EVs-NR and EVs-DOX

The cells were seeded at a density of 2.5 × 10^4^ cells per sterile round coverslip, placed in a 24-well plate, and cultured in 400 µL of McCoy’s 5A or MEM medium under standard conditions for 24 h. Then, the medium was discarded, the cells were gently washed with PBS, and 400 µL of Nile Red-stained EV or doxorubicin-loaded EV suspension in McCoy’s 5A or MEM medium (2.8 × 10^9^ EVs/mL, equivalent of 4.5 × 10^4^ EVs/cell) was added. As a negative control, nontreated cells or cells incubated with pure EVs of the same amount (400 μL of 2.8 × 10^9^ EVs/mL) were used. As a positive control for doxorubicin loading, 400 µL of free doxorubicin (0.45 mM) was added to wells. The cells were cultured for an additional 24 h under standard conditions. After that, the cells were washed with PBS, fixed with 3.7% paraformaldehyde (15 min, RT, dark), and washed again with PBS. The next step was staining nuclei with Hoechst 33342 (Invitrogen, Carlsbad, CA, USA) diluted in PBS (final concentration 0.5 μg/mL, 10 min, RT, dark). The dye was removed by washing with PBS (2 times), and coverslips were removed from the well and placed upside-down on a microscope slide. The observations were conducted with the use of a fluorescence microscope (Nikon Eclipse Ni) in white (DIC), blue (filter block FF01-392/23 nm excitation, FF02-447/60 nm emission), and green light (filter block FF01-474/27 nm excitation, FF02-525/45 nm emission) at 600× magnification (Nikon, Plan Fluor objective lens 60×/0.85 ∞/0.11–0.23 WD 0.40–0.31 B, 10× eyepiece).

### 3.10. Isolation of Proteins from EVs

Two sets of 400 μL samples of EVs collected from three independent cultures of *S. boulardii* were mixed with 100 μL of 100% TCA (trichloroacetic acid, Sigma-Aldrich) and incubated at 4 °C, first with vortexing for 10 min, and subsequently, stationary for an additional 30 min. Next, the samples were centrifuged (15,000× *g*, 10 min, 4 °C) to precipitate proteins, and the supernatants were discarded. Protein pellets were washed with 200 μL of cold acetone (4 °C) and centrifuged (15,000× *g*, 5 min, 4 °C), and the supernatants were discarded. The washing step was repeated 3 times in total to remove residual TCA. Finally, the protein pellets were dried for a few minutes at 37 °C to remove residual acetone. After that, one set of precipitates was subjected to determination of the protein concentration by applying Bradford’s reagent (Sigma-Aldrich) and BSA as a standard (BIO-RAD, Hercules, CA, USA). The second set of proteins precipitated from EVs was used for mass spectrometry analysis.

### 3.11. Mass Spectrometry (MS) Analysis and Profiling of EV Proteins

Precipitated proteins (61.40 ± 13.32 µg) from three independent EV samples were resuspended in 50 µL of 100 mM ammonium bicarbonate. Sample preparation and MS measurements were performed as described recently in [29]. After Evotip activation and equilibration, 20 μL of each sample was loaded onto the solid phase.

The raw data were preprocessed with Mascot Distiller software (v. 2.4.2.0; Matrix Science); then, the obtained peptide masses and fragmentation spectra were matched to the Saccharomyces Genome Database (SGD, 2016) using the Mascot search engine (Mascot Daemon v. 2.4.0 and Mascot Server v. 2.4.1, Matrix Science). Enzyme specificity was set to trypsin, peptide mass tolerance to 5 ppm, and fragment mass tolerance to 0.01 Da. The protein mass was left unrestricted with two missed cleavages allowed. Methylation of cysteine was set as fixed, and oxidation of methionine was set as a variable modification. Proteomic analysis was performed as described previously in [30]. The mass spectrometry proteomics data were deposited to the ProteomeXchange Consortium via the PRIDE [31] partner repository with the dataset identifier PXD042660 (http://www.ebi.ac.uk/pride/archive/projects/PXD042660; accessed on 29 June 2023). The list of identified proteins was exported to Microsoft Excell 2016 64-Bit Edition (Supplementary File: Table S1) and used for further bioinformatics analysis.

The proteins present in the three EV biological probes identified after matching to the SGD database (Supplementary File: Table S2) were analyzed in terms of their function in cellular processes with the use of ShinyGO 0.77 [32] based on the pathways in the KEGG Database (accessed 25 April 2023) [33].

### 3.12. Statistical Analysis

The data are presented as the means ± standard deviation (SD) from at least three independent experiments. Each biological test was performed three times. The statistically significant differences were analyzed by Student’s *t*-test with a statistical significance level of α = 5%. A *p*-value < 0.05 was considered statistically significant.

## 4. Conclusions

This study reports the EVs produced by the probiotic yeast *S. boulardii* CNCM I-745 and can be considered an introductory step of further research on their application as carriers of orally distributed biologically active substances. The mean size of isolated EVs was established at 127.39 ± 1.34 nm. In addition, it has been estimated that a single yeast cell produces at least 55 EVs. Both information about the size of EVs and the number of EVs released by the cells of *S. boulardii* are essential in view of their use as drug carriers.

This research has also shown that the tested vesicles are clearly not cytotoxic for three model human intestinal cell lines. In addition, yeast EVs are able to internalize in vitro with human cells and transfer their cargo. Moreover, the detailed proteomic analysis of these particles might be helpful for modifying the protein composition of yeast EVs so that they contain not only biologically active molecules but also receptor proteins that recognize, for example, cancer cells in colon tissues. This could lead to the specific transport of loaded biologically active substances to selected cells and tissues in the body. Taking all of the above into account, the final conclusion of the study is that probiotic yeast EVs are promising candidates as carriers of biologically active substances.

## Data Availability

The MS datasets generated and analyzed during the current study are available under ProteomeXchange in the PRIDE repository (https://www.ebi.ac.uk/pride/), with identifier PXD042660 (accessed on 29 June 2023).

## Supplementary Materials

The following supporting information can be downloaded at: https://www.mdpi.com/article/10.3390/ijms241411340/s1.

## References

[1] Freitas M.S., Bonato V.L.D., Pessoni A.M., Rodrigues M.L., Casadevall A., Almeida F. (2019). Fungal Extracellular Vesicles as Potential Targets for Immune Interventions. mSphere.

[2] Jung E.H., Park Y.-D., Dragotakes Q., Ramirez L.S., Smith D.Q., Reis F.C.G., Dziedzic A., Rodrigues M.L., Baker R.P., Williamson P.R. (2022). *Cryptococcus neoformans* releases proteins during intracellular residence that affect the outcome of the fungal–macrophage interaction. Microlife.

[3] Martínez-López R., Hernáez M.L., Redondo E., Calvo G., Radau S., Pardo M., Gil C., Monteoliva L. (2022). *Candida albicans* Hyphal Extracellular Vesicles Are Different from Yeast Ones, Carrying an Active Proteasome Complex and Showing a Different Role in Host Immune Response. Microbiol. Spectr..

[4] Rizzo J., Rodrigues M.L., Janbon G. (2020). Extracellular Vesicles in Fungi: Past, Present, and Future Perspectives. Front. Cell. Infect. Microbiol..

[5] Liebana-Jordan M., Brotons B., Falcon-Perez J.M., Gonzalez E. (2021). Extracellular Vesicles in the Fungi Kingdom. Int. J. Mol. Sci..

[6] Bielska E., May R.C. (2019). Extracellular vesicles of human pathogenic fungi. Curr. Opin. Microbiol..

[7] Mencher A., Morales P., Valero E., Tronchoni J., Patil K.R., Gonzalez R. (2020). Proteomic characterization of extracellular vesicles produced by several wine yeast species. Microb. Biotechnol..

[8] Agarbati A., Canonico L., Marini E., Zannini E., Ciani M., Comitini F. (2020). Potential Probiotic Yeasts Sourced from Natural Environmental and Spontaneous Processed Foods. Foods.

[9] Sen S., Mansell T.J. (2020). Yeasts as probiotics: Mechanisms, outcomes, and future potential. Fungal Genet. Biol..

[10] Navarro-López V., Hernández-Belmonte A., Pérez Soto M.I., Ayo-González M., Losa-Rodríguez G., Ros-Sánchez E., Martínez-Gabarrón M., Sánchez-Pellicer P., Aguera-Santos J., Núñez-Delegido E. (2022). Oral intake of *Kluyveromyces marxianus* B0399 plus *Lactobacillus rhamnosus* CECT 30579 to mitigate symptoms in COVID-19 patients: A randomized open label clinical trial. Med. Microecol..

[11] Moré M.I., Swidsinski A. (2015). *Saccharomyces boulardii* CNCM I-745 supports regeneration of the intestinal microbiota after diarrheic dysbiosis—A review. Clin. Exp. Gastroenterol..

[12] Arevalo-Villena M., Briones-Perez A., Corbo M.R., Sinigaglia M., Bevilacqua A. (2017). Biotechnological application of yeasts in food science: Starter cultures, probiotics and enzyme production. J. Appl. Microbiol..

[13] Pothoulakis C. (2009). Review article: Anti-inflammatory mechanisms of action of *Saccharomyces boulardii*. Aliment. Pharmacol. Ther..

[14] Domínguez Rubio A.P., D’antoni C.L., Piuri M., Pérez O.E. (2022). Probiotics, Their Extracellular Vesicles and Infectious Diseases. Front. Microbiol..

[15] Higuchi A., Morishita M., Nagata R., Maruoka K., Katsumi H., Yamamoto A. (2023). Functional Characterization of Extracellular Vesicles from Baker’s Yeast *Saccharomyces Cerevisiae* as a Novel Vaccine Material for Immune Cell Maturation. J. Pharm. Sci..

[16] Edwards-Ingram L., Gitsham P., Burton N., Warhurst G., Clarke I., Hoyle D., Oliver S.G., Stateva L. (2007). Genotypic and physiological characterization of *Saccharomyces boulardii*, the probiotic strain of *Saccharomyces cerevisiae*. Appl. Environ. Microbiol..

[17] Černoša A., Gostinčar C., Lavrin T., Kostanjšek R., Lenassi M., Gunde-Cimerman N. (2022). Isolation and characterization of extracellular vesicles from biotechnologically important fungus *Aureobasidium pullulans*. Fungal Biol. Biotechnol..

[18] Kulig K., Karnas E., Woznicka O., Kuleta P., Zuba-Surma E., Pyza E., Osyczka A., Kozik A., Rapala-Kozik M., Karkowska-Kuleta J. (2022). Insight Into the Properties and Immunoregulatory Effect of Extracellular Vesicles Produced by *Candida glabrata*, *Candida parapsilosis*, and *Candida tropicalis* Biofilms. Front. Cell. Infect. Microbiol..

[19] Juan C.A., Pérez de la Lastra J.M., Plou F.J., Pérez-Lebeña E. (2021). The Chemistry of Reactive Oxygen Species (ROS) Revisited: Outlining Their Role in Biological Macromolecules (DNA, Lipids and Proteins) and Induced Pathologies. Int. J. Mol. Sci..

[20] Sobiepanek A., Paone A., Cutruzzolà F., Kobiela T. (2021). Biophysical characterization of melanoma cell phenotype markers during metastatic progression. Eur. Biophys. J..

[21] Yeung T.M., Gandhi S.C., Wilding J.L., Muschel R., Bodmer W.F. (2010). Cancer stem cells from colorectal cancer-derived cell lines. Proc. Natl. Acad. Sci. USA.

[22] Simon H.-U., Haj-Yehia A., Levi-Schaffer F. (2000). Role of reactive oxygen species (ROS) in apoptosis induction. Apoptosis.

[23] Zhao K., Bleackley M., Chisanga D., Gangoda L., Fonseka P., Liem M., Kalra H., Al Saffar H., Keerthikumar S., Ang C.-S. (2019). Extracellular vesicles secreted by *Saccharomyces cerevisiae* are involved in cell wall remodelling. Commun. Biol..

[24] Oliveira D.L., Nakayasu E.S., Joffe L.S., Guimarães A.J., Sobreira T.J.P., Nosanchuk J.D., Cordero R.J.B., Frases S., Casadevall A., Almeida I.C. (2010). Characterization of Yeast Extracellular Vesicles: Evidence for the Participation of Different Pathways of Cellular Traffic in Vesicle Biogenesis. PLoS ONE.

[25] Gil-Bona A., Llama-Palacios A., Parra C.M., Vivanco F., Nombela C., Monteoliva L., Gil C. (2015). Proteomics unravels extracellular vesicles as carriers of classical cytoplasmic proteins in *Candida albicans*. J. Proteome Res..

[26] Ruzycka-Ayoush M., Nowicka A.M., Kowalczyk A., Gluchowska A., Targonska A., Mosieniak G., Sobczak K., Donten M., Grudzinski I.P. (2023). Exosomes derived from lung cancer cells: Isolation, characterization, and stability studies. Eur. J. Pharm. Sci..

[27] Sobiepanek A., Milner-Krawczyk M., Musolf P., Starecki T., Kobiela T. (2022). Anandamide-Modulated Changes in Metabolism, Glycosylation Profile and Migration of Metastatic Melanoma Cells. Cancers.

[28] Lennaárd A.J., Mamand D.R., Wiklander R.J., EL Andaloussi S., Wiklander O.P.B. (2021). Optimised Electroporation for Loading of Extracellular Vesicles with Doxorubicin. Pharmaceutics.

[29] Kamińska K., Godakumara K., Świderska B., Malinowska A., Midekessa G., Sofińska K., Barbasz J., Fazeli A., Grzesiak M. (2023). Characteristics of size-exclusion chromatography enriched porcine follicular fluid extracellular vesicles. Theriogenology.

[30] Sulkowska A., Auber A., Sikorski P.J., Silhavy D.N., Auth M., Sitkiewicz E., Jean V., Merret R.M., Bousquet-Antonelli C.C., Kufel J. (2020). RNA Helicases from the DEA(D/H)-Box Family Contribute to Plant NMD Efficiency. Plant Cell Physiol..

[31] Perez-Riverol Y., Bai J., Bandla C., García-Seisdedos D., Hewapathirana S., Kamatchinathan S., Kundu D.J., Prakash A., Frericks-Zipper A., Eisenacher M. (2021). The PRIDE database resources in 2022: A hub for mass spectrometry-based proteomics evidences. Nucleic Acids Res..

[32] Ge S.X., Jung D., Yao R. (2020). ShinyGO: A graphical gene-set enrichment tool for animals and plants. Bioinformatics.

[33] Kanehisa M., Furumichi M., Sato Y., Kawashima M., Ishiguro-Watanabe M. (2023). KEGG for taxonomy-based analysis of pathways and genomes. Nucleic Acids Res..

